# Ubiquitin-Specific Protease 25 Functions in Endoplasmic Reticulum-Associated Degradation

**DOI:** 10.1371/journal.pone.0036542

**Published:** 2012-05-09

**Authors:** Jessica R. Blount, Aaron A. Burr, Amanda Denuc, Gemma Marfany, Sokol V. Todi

**Affiliations:** 1 Department of Pharmacology and Department of Neurology, Wayne State University School of Medicine, Detroit, Michigan, United States of America; 2 Graduate Program in Cancer Biology, Wayne State University School of Medicine, Detroit, Michigan, United States of America; 3 Department of Genetics, Faculty of Medicine, University of Barcelona, Barcelona, Spain; Centro de Biología Molecular Severo Ochoa (CSIC-UAM), Spain

## Abstract

Endoplasmic Reticulum (ER)-associated degradation (ERAD) discards abnormal proteins synthesized in the ER. Through coordinated actions of ERAD components, misfolded/anomalous proteins are recognized, ubiquitinated, extracted from the ER and ultimately delivered to the proteasome for degradation. It is not well understood how ubiquitination of ERAD substrates is regulated. Here, we present evidence that the deubiquitinating enzyme Ubiquitin-Specific Protease 25 (USP25) is involved in ERAD. Our data support a model where USP25 counteracts ubiquitination of ERAD substrates by the ubiquitin ligase HRD1, rescuing them from degradation by the proteasome.

## Introduction

Protein quality control consists of basic cellular pathways necessary for homeostasis. Malfunctions in protein quality control are linked to malignancies, neurodegenerative diseases and metabolic syndromes [1]. In eukaryotic protein quality control most short-lived, abnormal proteins are recycled by the ubiquitin-proteasome system: proteins that need to be discarded are selectively ubiquitinated and the poly-ubiquitin chain is ultimately recognized by the proteasome for degradation. Post-translational modification of proteins by ubiquitin is accomplished through the concerted action of three enzymes. The ubiquitin-activating enzyme (E1) activates ubiquitin and transfers it to a ubiquitin-conjugating enzyme (E2). In the presence of a ubiquitin ligase (E3), ubiquitin is transferred most commonly to a lysine residue of a substrate protein. Like many other types of post-translational modifications, ubiquitination is reversible. Indeed, deubiquitination is critical for normal cell function and is accomplished by deubiquitinating enzymes (DUBs) [2], [3]. The human genome encodes nearly 90 DUBs [4], several of which have been linked to protein quality control [2], [3], [5].

One function of the ubiquitin-proteasome system is to degrade luminal or trans-membrane peptides that are produced in the endoplasmic reticulum (ER) [1], [6]. During ER-Associated Degradation (ERAD), misfolded proteins are recognized, deglycosylated, ubiquitinated, extracted into the cytosol and ultimately presented to the proteasome for degradation [6]. Each step is conducted by protein complexes that are recruited and assembled around proteins that need to be degraded. HRD1 is one of several ER-resident ubiquitin ligases involved in ubiquitination [7]–[9]. Ubiquitination of ERAD substrates is coupled to their extraction from the ER into the cytosol by the AAA ATPase VCP/p97. Substrate ubiquitination appears necessary for extraction [6]. VCP/p97 is brought to the ER membrane by cofactors that recognize ubiquitin chains on ERAD substrates [1], [6]. Following extraction, substrates are escorted to the proteasome for degradation. Despite significant advances in understanding individual steps in ERAD [1], [6], [10]–[13] and evidence of at least three DUBs involved in this pathway (USP19, ataxin-3 and YOD1 [14]–[17]) it is not entirely clear how substrate ubiquitination is regulated during ERAD.

Ubiquitin-Specific Protease 25 (USP25) is a catalytically active DUB *in vitro* [18], [19], previously reported to regulate proteasomal turnover of muscle proteins [18]. Here, we present evidence that USP25 functions in ERAD. USP25 interacts with HRD1 and VCP/p97 and rescues several ERAD substrates from degradation by the proteasome. Our work sheds light on a previously unknown ERAD component.

## Results

### USP25 localizes at the ER and interacts with ERAD components

**Figure 1:**
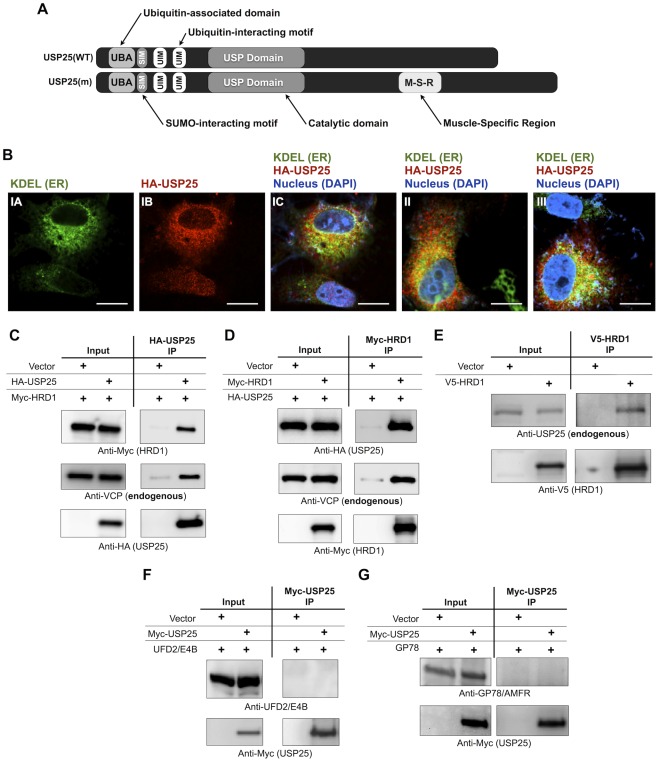
USP25 interacts with ERAD components. A) Schematics depict known domains of common (USP25(WT)) and muscle-specific (USP25(m)) isoforms of USP25 that are expressed in mammals [18], [19], [41], [42]. B) HEK-293 cells were transfected with HA-USP25. 48 hours later cells were fixed, probed as indicated and imaged with laser confocal microscopy. Panels IA-IC are single optical plane images (1 µM) of a cell immunolabeled for ER (KDEL, endogenous marker), HA-USP25 and nucleus (DAPI). Panel IC is the merged view of panels IA (green channel), IB (red channel) and DAPI (blue channel; not shown as a separate channel). Panels II and III are merged views of other cells stained similarly to panel I. Scale bars: 10 µM. C–G) HEK-293 cells were transfected as shown. Indicated constructs were immunopurified with bead-bound antibodies. Similar results were obtained from COS-7 cells for panels B–E (not shown). All USP25 constructs used in this figure were the common isoform (USP25(WT)).

The two isoforms of the deubiquitinating enzyme USP25 (Figure 1A), according to a previous report, have a cellular distribution somewhat reminiscent of ER staining [20]. Therefore, we conducted confocal microscopy with an endogenous ER marker. As shown in figure 1B, some USP25 localizes at the ER. We consequently examined whether USP25 interacts with ERAD components. By conducting co-immunoprecipitation experiments from cells, we found that exogenous USP25 interacts with the ER-resident ubiquitin ligase HRD1 and with endogenous VCP/p97 (Figure 1C). Conversely, HRD1 interacts with USP25 and VCP/p97 (Figure 1D). Importantly, HRD1 and endogenous USP25 interact in cells (Figure 1E), but USP25 does not interact with other ubiquitin ligases implicated in ERAD [6], [21]–[24]: UFD2/E4B (Figure 1F) and GP78/AMFR (Figure 1G). These results collectively demonstrate that USP25 interacts with some but not all ERAD components, suggesting a specific or selective interaction.

### USP25 regulates turnover of several ERAD substrates

**Figure 2 pone-0036542-g002:**
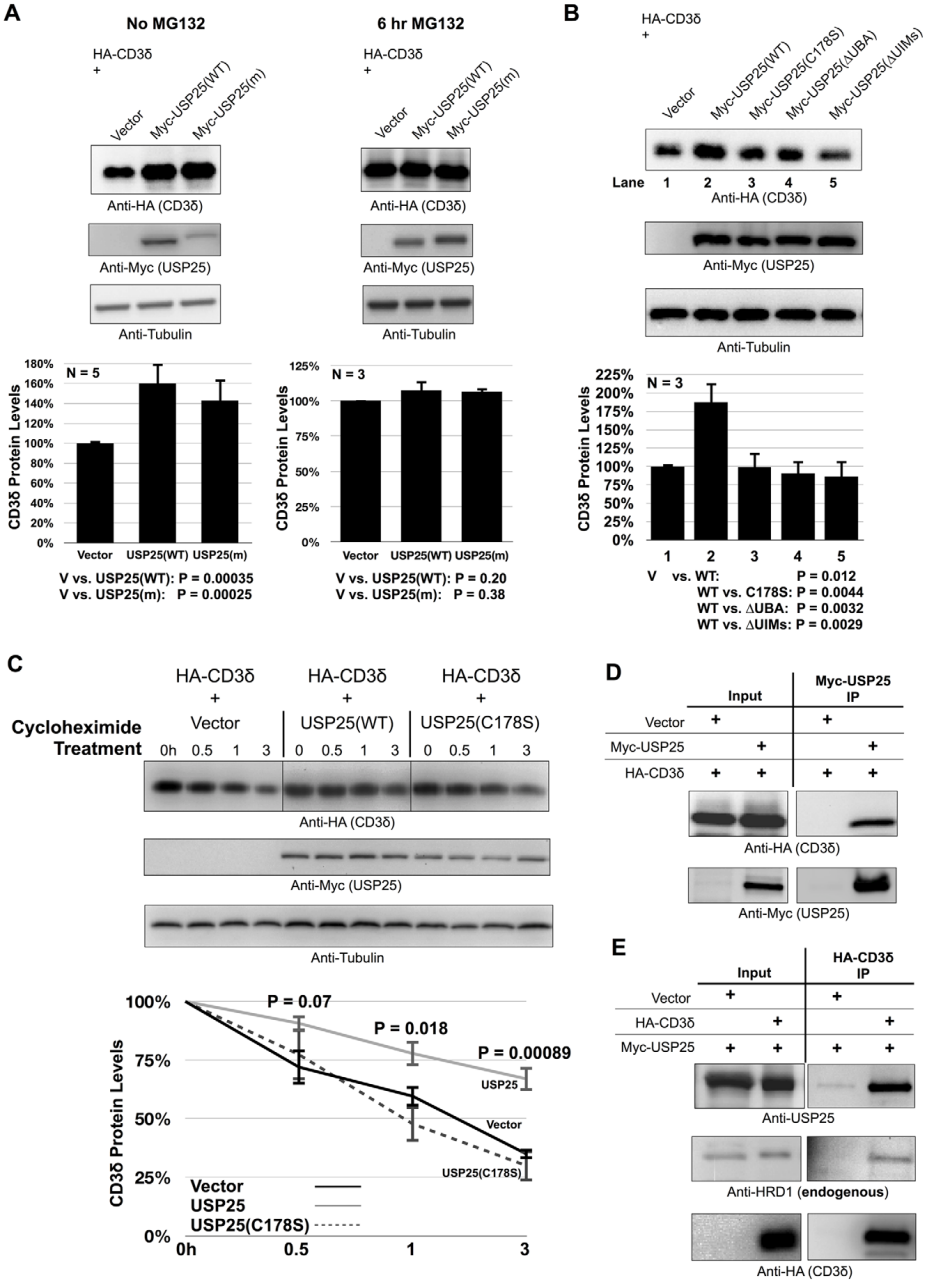
USP25 inhibits degradation of the ERAD substrate CD3δ . A) Western blots of whole cell lysates. Top: HEK-293 cells were transfected as indicated and treated with the proteasome inhibitor MG132 where noted (15 µM, 6 hours) before harvesting. Bottom: semi-quantification of bands from western blots shown above and other similar, independent experiments. CD3δ protein levels were normalized to loading control. Shown are means +/− standard deviations. USP25(WT): common isoform of USP25; USP25(m): muscle-specific isoform of USP25. P values from Student T-tests are shown below histograms. B) Top: HEK-293 cells were transfected with the indicated constructs and harvested 48 hours later. Shown are western blots of whole cell lysates probed with the indicated antibodies. WT: wild type USP25, C178S: the catalytic cysteine of USP25 was replaced by a serine residue [18], ΔUBA: UBA deleted, ΔUIM: both UIMs deleted. Bottom: semi-quantification of data from the top and two other independent experiments. CD3δ protein levels were normalized to loading control. Shown are means +/− standard deviations. P values from Student T-tests are shown below histograms. C) Top: HEK-293 cells were transfected as indicated. 48 hours post-transfection cells were treated for the indicated periods of time with 75 µg/ml cycloheximide to inhibit synthesis of new protein. Bottom: semi-quantification of western blots from the top and three other, independent experiments. CD3δ levels were normalized to loading control. Shown are means +/− standard deviations. P values are from Student T-tests of USP25 compared to vector control. D and E) HEK-293 cells were transfected with the indicated constructs. 48 hours later tagged constructs were immunopurified with bead-bound antibodies and probed as indicated.

Since USP25 localizes at the ER and interacts with at least two ERAD components (Figure 1), we tested whether USP25 regulates protein levels of ERAD substrates. Both the common and muscle-specific isoforms of USP25 lead to higher steady state protein levels of the ERAD substrate CD3δ (Figure 2A, left panel). CD3δ is a trans-membrane subunit of the T cell receptor that in the absence of other subunits is degraded by the proteasome. USP25 seems to rescue CD3δ from proteasomal degradation, because after treatment with the proteasome inhibitor MG132 the effect of USP25 expression in stabilizing CD3δ protein is no longer observed (Figure 2A, right panel).

The catalytic activity of USP25 is necessary for its ability to increase steady state levels of CD3δ protein, as demonstrated by using a USP25 construct, C178S, where the catalytic cysteine is mutated into a serine residue (Figures 2B, 2C). Also, deleting either the ubiquitin-associated domain (UBA) or the ubiquitin-interacting motifs (UIMs) of USP25 abolishes the positive effect that USP25 has on the steady state levels of CD3δ protein (Figure 2B). Thus, the ability of USP25 to both bind and cleave ubiquitin appears to be required to exert the rescue effect on CD3δ.

To examine the effect of USP25 in the turnover of CD3δ protein, we co-transfected cells with CD3δ and either empty vector, wild type USP25 or catalytically inactive USP25 (C178S), then inhibited the translation of new protein with cycloheximide for pre-determined periods of time. As shown in figure 2C, USP25 significantly decelerates the degradation of CD3δ protein, increasing its half-life. This effect depends on the catalytic activity of USP25, as catalytically inactive USP25 (C178S) does not alter the turnover of CD3δ protein (Figure 2C). The cycloheximide-based approach we used here is based on semi-quantification of western blots. Although non-linear, this assay provides valuable clues on the half-life and rates of protein turnover, supporting the notion that catalytically active USP25 slows down the degradation of CD3δ protein.

Next, we examined whether USP25 and CD3δ interact in cells. Panels 2D and E show that USP25 and CD3δ co-immunoprecipitate each other from cells. CD3δ also co-immunoprecipitates endogenous HRD1 alongside USP25 (Figure 2E), suggesting that HRD1 and USP25 might co-regulate CD3δ, forming part of the same regulatory post-translational modification complex.

**Figure 3:**
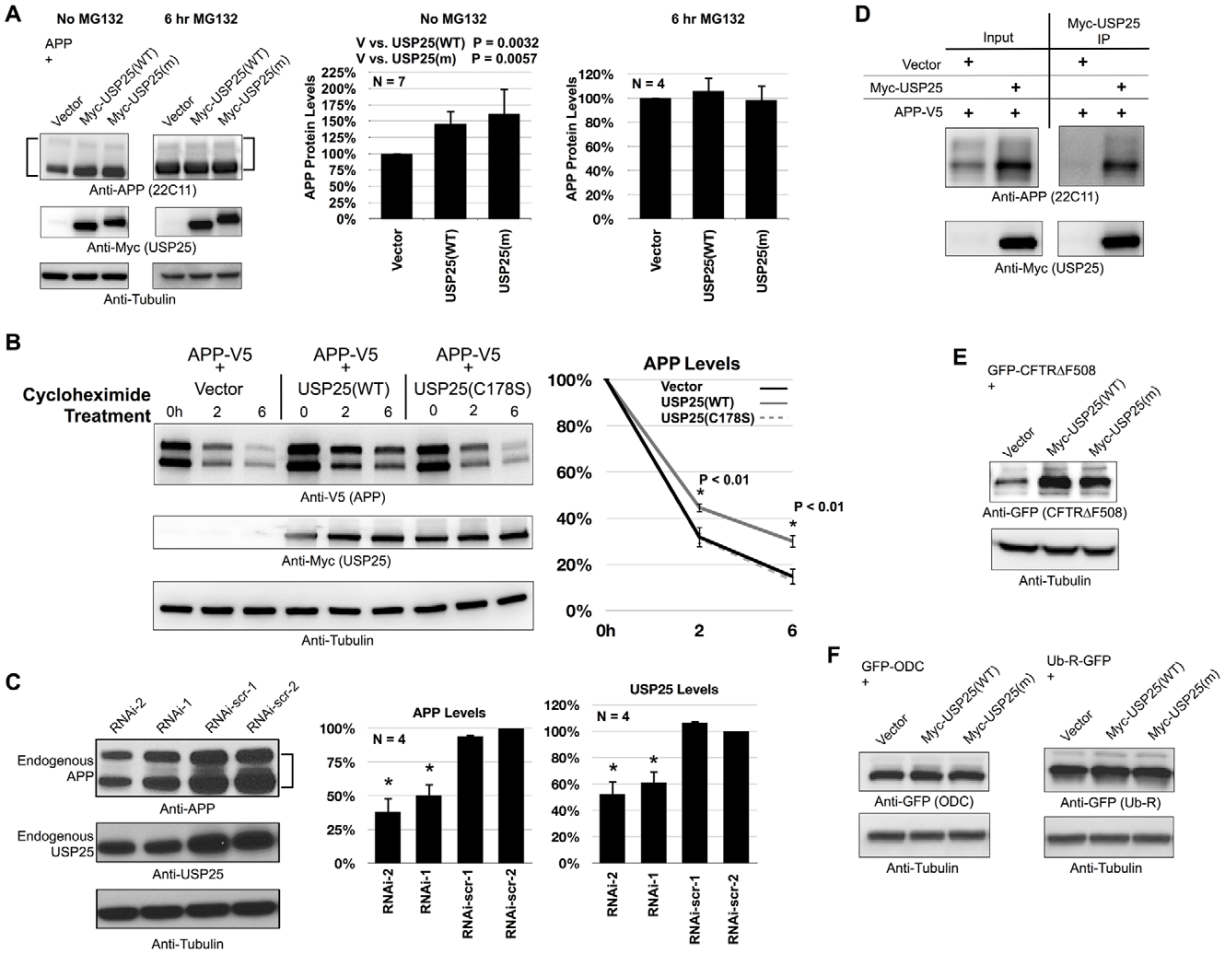
USP25 regulates protein levels of the ERAD substrates APP and CFTRΔF508. A) Left: whole cell lysates of HEK-293 cells transfected with the indicated constructs. USP25 (WT) and USP25(m) are both catalytically active isoforms. Where noted, cells were treated with the proteasome inhibitor MG132 (15 µM, 6 hrs) before harvesting. Right: histograms show semi-quantification of APP signal from the left portion and other similar, independent experiments. Bracket: APP bands were quantified separately, added and normalized to loading control. Shown are means +/− standard deviations. P values from Student T-tests are shown above histograms. No statistically significant differences were observed when cells were treated with MG132. B) Left: whole cells lysates of HEK-293 cells transfected as indicated and treated 48 hours later with cycloheximide to inhibit translation of new protein. Right: semi-quantification of western blots from the right and two other independent experiments. Shown are means +/− standard deviations. APP levels were normalized to loading control. P values are from Student T-tests where APP levels in the presence of USP25(WT) were compared to APP levels in presence of vector control. C) Left: HEK-293 cells were transfected with shRNA constructs targeting different portions of endogenous USP25 (RNAi-1, 2) or scramble RNA (RNAscr-1, 2). Cells were harvested 48 hours post-transfection and probed as indicated in western blots. Trials with 72 hour-long transfections yielded similar results (not shown). Right: semi-quantification of signal from the left and other similar, independent experiments. Bracket: APP bands were quantified separately, added together and normalized to loading control. Asterisks: P<0.01 according to Student T-tests comparing RNAi-1 and RNAi-2 lanes to RNAi-scr lanes. D) HEK-293 cells were transfected with the indicated constructs and Myc-USP25 was co-immunoprecipitated 48 hours later. E and F) HEK-293 cells were transfected with the indicated constructs. Western blots of whole cell lysates. For panels D, E and F: similar results were obtained from COS-7 cells (not shown).

Another ERAD substrate is β-Amyloid Precursor Protein (APP), whose turnover is reportedly regulated by HRD1 [25]. Catalytically active isoforms of USP25 lead to moderately, but statistically significantly, higher steady state levels of APP protein in cells (Figure 3A) similar to this DUB's effect on the protein levels of CD3δ. The positive effect of USP25 on APP protein is detectable only when the proteasome is active; treatment of transfected cells with the inhibitor MG132 abolishes this effect (Figure 3A), suggesting that USP25 rescues APP from proteasomal degradation. In cycloheximide-based time course experiments, where production of new protein is halted, USP25 modestly, but statistically significantly, slows down APP turnover (Figure 3B). Similarly to what occurs with CD3δ, overexpression of USP25 increases APP half-life. Again, this effect depends on the catalytic activity of USP25, as catalytically inactive USP25(C178S) does not alter APP turnover (Figure 3B).

On a side note, when comparing untreated cells to ones treated with proteasome inhibitor, the muscle-specific isoform of USP25 (USP25(m)) appears less stable than its common counterpart (USP25(WT)) in some cases (Figure 2A), but not so much in others (Figure 3A). The basis of this difference is unclear. Perhaps the stability of the USP25(m) isoform depends in part on its co-expressed partners.

Since HEK-293 cells express APP endogenously, we approached the rescue effect of USP25 upon APP protein under more physiological conditions, without over-expression of USP25. We tested whether RNAi-mediated knockdown of endogenous USP25 causes a reduction in endogenous APP protein levels. We used 48- and 72-hour long transfection periods and tested seven different shRNA constructs, but achieved only approximately 50% reduction in endogenous USP25 protein (Figure 3C and data not shown; see Materials and Methods). Still, even modest knockdown of endogenous USP25 leads to significantly lower steady state levels of endogenous APP protein (Figure 3C). Since APP and USP25 also co-immunoprecipitate from cells (Figure 3D), our results collectively suggest that USP25 regulates APP protein degradation.

Although our data relate USP25 to ERAD substrate turnover (Figures 1–3), we nevertheless wanted to address the possibility that USP25 acts non-specifically on all proteasomal targets, including both ERAD non-ERAD substrates. We examined the effect of USP25 on steady state levels of yet another ERAD substrate, CFTRΔF508 [6], and two non-ERAD substrates, Ub-R-GFP (N-End rule degradation; [26]) and GFP-ODC (proteasomal degradation independent of a poly-ubiquitin signal; [27]). USP25 increases the steady state protein levels of the ERAD substrate CFTRΔF508 (Figure 3E), but does not affect either non-ERAD substrate (Figure 3F). Previous work also found that USP25 does not have a general effect on proteasomal targets, as assessed by general ubiquitination patterns in cells [20]. Therefore, our findings implicate USP25 more specifically in ERAD substrate turnover, at least for some ERAD substrates.

### USP25 opposes the effect of HRD1 on CD3δ in cells

**Figure 4:**
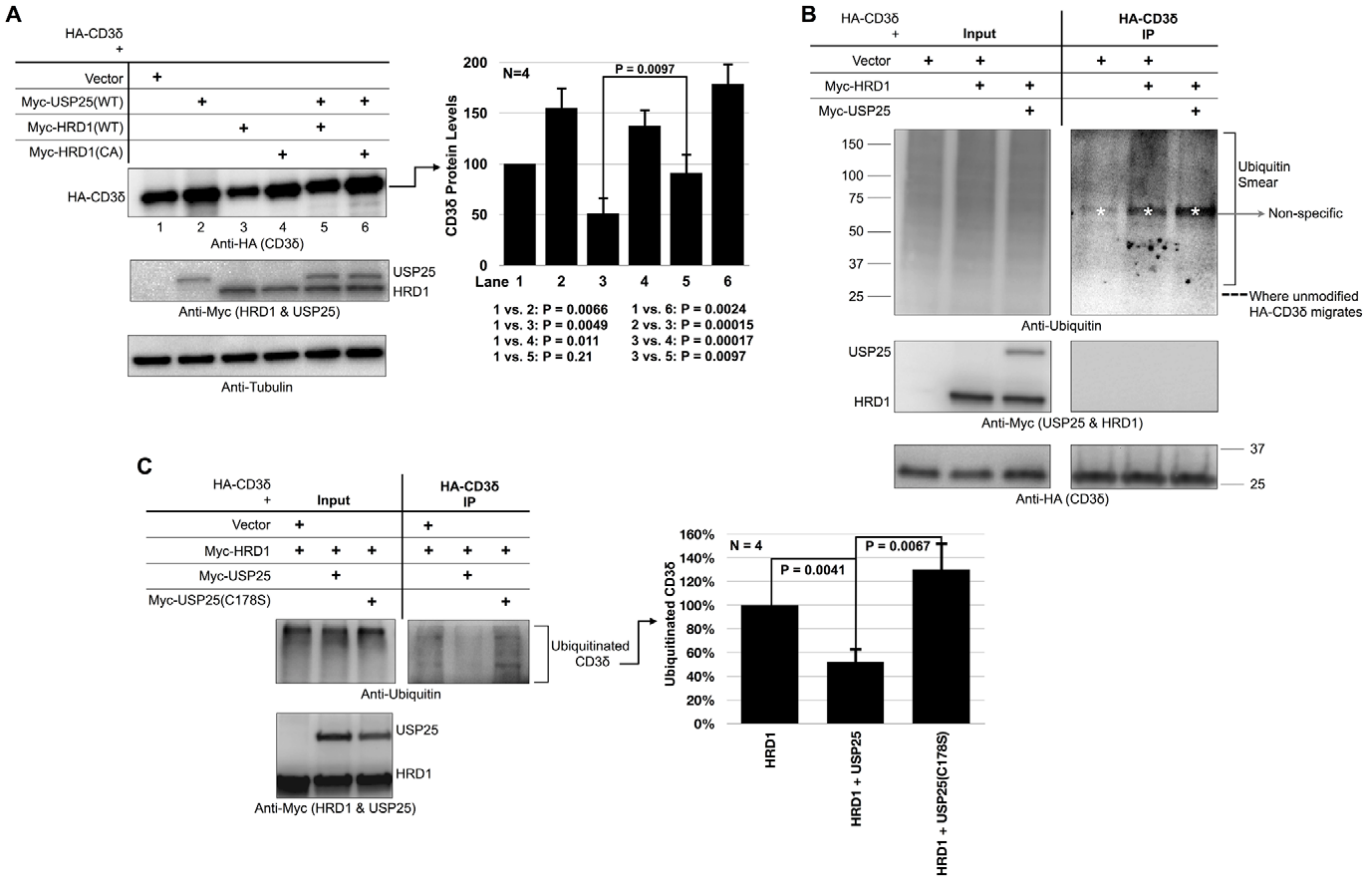
USP25 and HRD1 have opposing effects on CD3 δ **protein levels and ubiquitination.** A) HEK-293 cells were transfected as indicated and harvested 48 hours later. Western blots are from whole cell lysates. HRD1(WT): normal HRD1; HRD1(CA): catalytically inactive HRD1, in which the catalytic cysteine is substituted by an alanine residue [7]. Histograms on the right: semi-quantification of data from the left and other independent experiments. Shown are means +/− standard deviations. CD3δ levels were normalized to loading control. P values from Student T tests are shown below histograms. B and C) HEK-293 cells were transfected with the indicated constructs. 48 hours post transfection, cells were treated for 6 hours with MG132 (15 µM) and HA-CD3δ was immunopurified using bead-bound anti-HA antibody after a stringent denature/renature step (see Materials and Methods for details). Histograms: semi-quantification of bracketed ubiquitin smears from the experiment on the left and other similar, independent experiments. Shown are means +/− standard deviations. P values for panel C are from Student T-tests.

CD3δ interacts with both USP25 and HRD1 in cells (Figure 2E), suggesting a functional interaction between HRD1 and USP25 in ERAD. Therefore, we examined the combinatorial effect of HRD1 and USP25 on steady state levels of CD3δ protein through co-transfection experiments. HRD1 decreases steady state protein levels of CD3δ in a manner dependent on its catalytic activity (lanes 1, 3 and 4 in Figure 4A), as reported previously [7]. The positive effect of HRD1 on CD3δ degradation is reversed by wild type USP25, whose co-expression causes an increase in CD3δ protein levels (compare lanes 3 and 5 in Figure 4A). These data support a model where USP25 counteracts the ubiquitin ligase function of HRD1. Consequently, we investigated the effect of HRD1 and USP25 on CD3δ ubiquitination and its presumed targeting to the proteasome for degradation, by using established, stringent purification protocols to isolate CD3δ from cells ([28]–[31]; see Materials and Methods). As shown in figure 4B, HRD1 increases ubiquitination of CD3δ in cells. The presence of USP25 significantly reduces levels of ubiquitinated CD3δ (Figure 4B, C). Notably, over-expression of catalytically inactive USP25(C178S) does not have a noticeable effect on CD3δ protein levels, its rate of degradation or ubiquitination (Figure 2 and Figure 4C). These results together with the interaction between CD3δ and USP25 (Figure 2), suggest that USP25 might rescue CD3δ from proteasomal degradation by directly deubiquitinating it.

### USP25 decreases ubiquitination of endogenous proteins associated with HRD1 and VCP/p97

**Figure 5:**
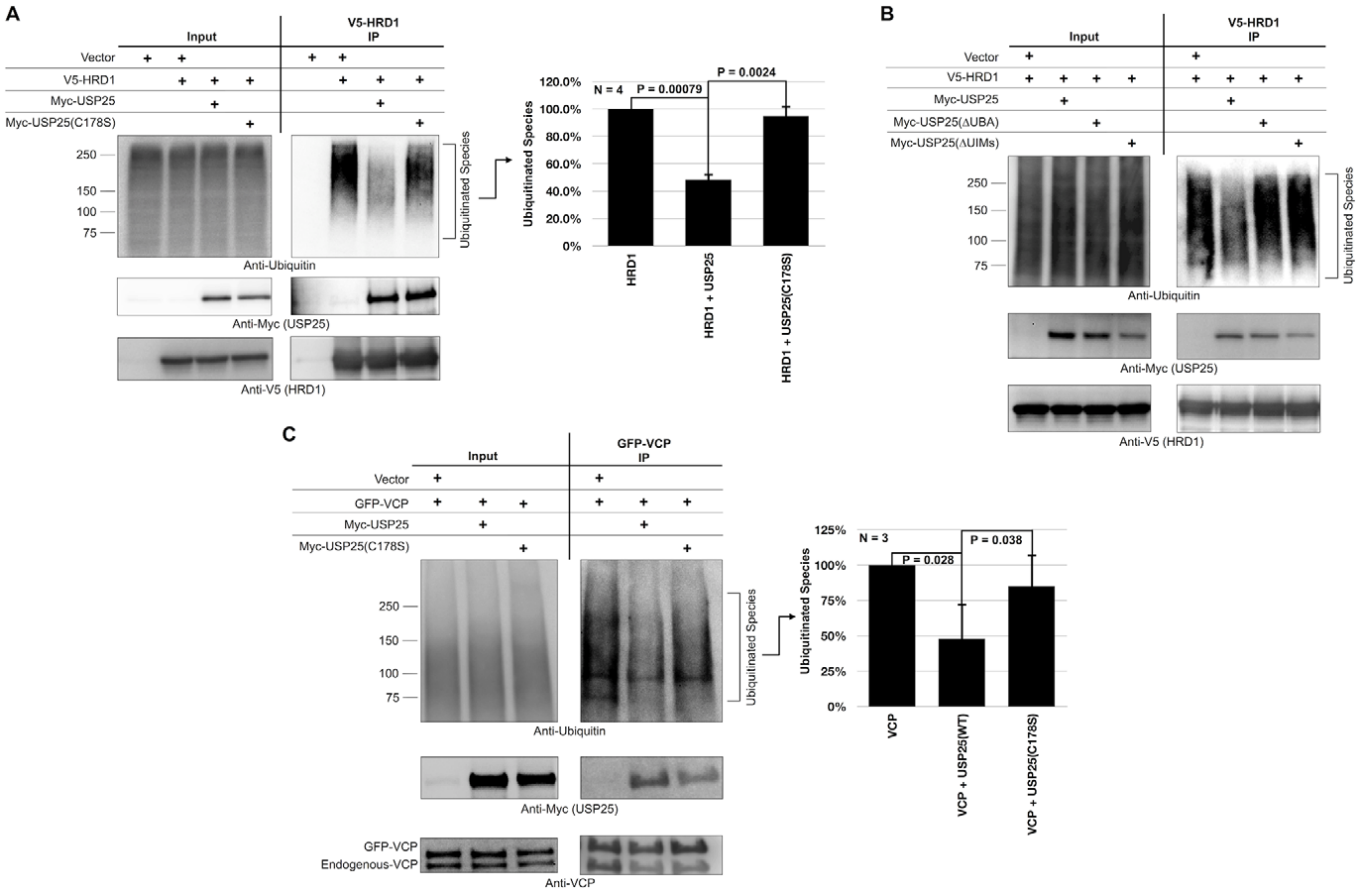
USP25 reduces levels of ubiquitinated endogenous proteins associated with HRD1 or VCP/p97. A–C) HEK-293 cells were transfected as indicated. 48 hours later cells were harvested and constructs were immunopurified with bead-bound anti-V5 antibody to isolate V5-HRD1-associated protein (panels A and B), or with bead-bound anti-GFP antibody to isolate GFP-VCP-associated proteins (panel C). We did not detect ubiquitin species in western blots from stringent, denature/renature immunoprecipitations of HRD1 or VCP/p97 using RIPA buffer (data not shown), indicating that the ubiquitin signal we observe in this figure is due to species associated with HRD1 or VCP/p97. Shown are western blots of inputs from whole cells lysates and from immunopurified protein. Histograms are semi-quantifications of results from the left and other similar, independent experiments. Shown are means +/− standard deviations. P values for panels A and C are from Student-T tests.

We next examined the effect of USP25 on the ubiquitination status of endogenous proteins associated with HRD1 and their presumptive targeting to the proteasome. We immunopurified endogenous proteins associated with transfected HRD1 from cells in the absence or presence of co-transfected USP25, following previously published protocols ([16], [17]; see Materials and Methods). As shown in figure 5A, USP25 significantly reduces levels of endogenous ubiquitinated species that co-immunoprecipitate with HRD1. Importantly, the effect of USP25 on lowering levels of ubiquitinated species that co-immunoprecipiate with HRD1 depends on the catalytic activity and ubiquitin-binding domains of USP25: neither catalytically inactive USP25(C178S) (Figure 5A) nor USP25 with deleted UBA or UIMs (Figure 5B) have the same effect. These results further suggest that USP25 counteracts the ubiquitin ligase function of HRD1 by binding ubiquitinated species and cleaving them. Lastly, neither the UBA nor the UIMs of USP25 appear important for its ability to interact with HRD1, since HRD1 interacts with USP25 lacking either domain (Figure 5B).

Because USP25 also interacts with endogenous VCP/p97 (Figure 1), we investigated the effect of USP25 on the ubiquitination status of endogenous proteins associated with VCP/p97. As shown in figure 5C, transfected USP25 reduces ubiquitination of endogenous species associated with transfected and endogenous VCP/p97. However, the effect of USP25 on VCP/p97 ubiquitinated clients is not as statistically robust as that on HRD1-associated species. Collectively, this work suggests that USP25 has a general inhibitory effect on ERAD substrate turnover, particularly on HRD1 substrates.

## Discussion

Regulation of ubiquitin-dependent mechanisms in ERAD is not fully understood. Here, we presented evidence that the DUB USP25 is a component of ERAD. USP25 joins other DUBs involved in ERAD: ataxin-3 [16], [17], YOD1 [14] and USP19 [15]. USP19 is a trans-membrane DUB on the cytoplasmic face of the ER membrane, where it reportedly rescues ERAD substrates through deubiquitination [15]. It is unclear with which ERAD components USP19 interacts functionally. Ataxin-3 and YOD1 are involved in ERAD by binding directly to VCP/p97. Their DUB activity appears to mediate delivery of ERAD substrates to the proteasome by VCP/p97 [14], [16], [17].

According to our results, USP25: a) interacted with and rescued the ERAD substrates CD3δ and APP, and counteracted HRD1 effects on CD3δ, b) localized in part at the ER and interacted with the ERAD components HRD1 and VCP/p97, c) reduced the levels of endogenous ubiquitinated species associated with HRD1 and VCP/p97, and d) regulated the levels of endogenous APP, as knockdown of endogenous USP25 was associated with lower levels of endogenous APP. Based on these data, we propose that USP25 deubiquitinates ERAD substrates while or after they are ubiquitinated by HRD1, rescuing them from proteasomal degradation. The catalytic activity and ubiquitin-binding domains of USP25 appear necessary both to rescue ERAD substrates and to lower the levels of HRD1-associated ubiquitinated species, suggesting that USP25 must bind ubiquitin chains in order to cleave them. According to previous reports, CFTRΔF508 is not an HRD1 substrate [6]. Therefore, our results where USP25 increased CFTRΔF508 protein levels suggest that USP25 could also function in ERAD independently of HRD1. However, we did not observe an interaction between USP25 and other ligases implicated in ERAD, GP78/AMFR and UFD2/E4B.

Our model of USP25 action differs from that of YOD1 and ataxin-3. Through direct interactions with VCP/p97, ataxin-3 and YOD1 function after VCP/p97 is recruited to the ER membrane [14], [16], [17]. Once VCP/p97 is recruited to the ER membrane by ubiquitinated substrates, deubiquitination seems necessary for their extraction by VCP/p97 [6]. For example, catalytically inactive YOD1 leads to accumulation of ERAD substrates at a step preceding VCP/p97-dependent extraction [14], [32]. After successful extraction, substrates are presumably re-ubiquitinated by cytosolic ubiquitin ligases and finally escorted to the proteasome for degradation [6]. Since USP25 interacted with VCP/p97, USP25 might deubiquitinate ERAD substrates while they are bound by VCP/p97. However, if USP25 were to deubiquitinate substrates bound by VCP/p97, it would be expected to facilitate substrate extraction from the ER and enhance their subsequent degradation [14], [32]. Instead, we observed that USP25 rescued ERAD substrates and reduced levels of ubiquitination of HRD1-associated species. Our work suggests that USP25 functions during substrate ubiquitination at the ER membrane, before extraction by VCP/p97. Data in figure 5C, where lower levels of ubiquitinated species associated with VCP/p97 in the presence of USP25 were observed, could be due to fewer ERAD substrates available for recognition and extraction by VCP/p97.

It is unclear whether USP25-HRD1 or USP25-VCP/p97 interactions are direct. Sequence analyses did not identify areas indicative of a direct interaction between USP25 and VCP/p97 (not shown). Mammalian HRD1 does interact directly with VCP/p97 [33], which can explain our observation that USP25 co-precipitated VCP/p97. It needs to be determined how the complex that comprises USP25, HRD1, VCP/p97 and perhaps other components is precisely coordinated during ERAD.

E3 ligases and DUBs interact functionally to decide the fate of a protein [34]–[37]. The interaction between USP25 and HRD1 could serve as an editing step to help determine whether a protein should undergo ERAD. Functional interactions between DUBs (e.g. USP25) and ubiquitin ligases (e.g. HRD1) are probably dynamically monitored to ensure optimal recycling rates for either specific proteins or in-bulk degradation during protein quality control.

According to our results, USP25 altered the levels of APP. This finding may have implications for Alzheimer's Disease, because mutated forms of APP and higher levels of wild type APP are linked to Alzheimer's Disease [38]. Our observations that USP25 interacted with APP and affected APP turnover implicate USP25 in Alzheimer's Disease pathogenesis and may serve as a point of intervention for new therapeutic strategies. Future studies are necessary to understand the precise molecular mechanism of USP25 function in ERAD, as well as its potential implication in diseases associated to mutated and aggregated proteins, or faulty ERAD.

## Materials and Methods

### Constructs and antibodies

All constructs have been previously described [7], [17], [18], [26], [27], [39], [40]. For RNAi-mediated knockdown, two scrambled negative controls and seven USP25 shRNA constructs (V2LHS_63867, V2LHS_63830, V2LHS_63903, V2LHS_63904, V2LHS_63902, V2LHS_5201, V3LHS_310311) were purchased from Open Biosystems. Antibodies: mouse anti-APP, clone 22c11 from Millipore, was used at 1∶1000 dilution; mouse anti-Myc, clone 9e10 from Santa Cruz Biotech, was used at 1∶250 dilution; rabbit anti-HA, clone Y11 from Santa Cruz Biotech, was used at 1∶1000 dilution; mouse anti-V5 from Invitrogen, was used at 1∶5000 dilution; rabbit anti-ubiquitin from Dako, was used at 1∶500 dilution; rabbit anti-GFP from Santa Cruz Biotech, was used at 1∶1000 dilution; rabbit anti-VCP from Millipore, was used at 1∶5000 dilution; mouse anti-KDEL from Enzo Life Sciences, was used at 1∶500 dilution; rabbit anti-USP25 was described previously [18], [20], and was used at 1∶4000 dilution; mouse anti-E4B from BD Biosciences, was used at 1∶500 dilution; rabbit anti-GP78/AMFR from Cell Signaling, was used at 1∶1000 dilution; mouse anti-Tubulin from Sigma-Aldrich, was used at 1∶40000 dilution. Peroxidase-conjugated, secondary antibodies were from Jackson Immunoresearch, were used at 1∶15000 dilution.

### Cell transfections, imaging, densitometry and immunofluorescence

HEK-293 and COS-7 cell lines used in this study were purchased from ATCC and grown using standard conditions. Cells were transfected using Lipofectamine LTX (Invitrogen), following the manufacturer's instructions. 48 hours post-transfection, cells were harvested in 1% SDS/100mM DTT boiling lysis buffer for whole cell extracts. For RNAi-mediated knockdown, 72-hour transfections were also conducted, with similar results. Western blotting, digital imaging and densitometry were conducted as previously described [30], [31] using a CCD camera-equipped Bio-Rad Versadoc 5000MP imager. Images were all collected below saturation levels and background was removed uniformly before semi-quantification with Quantity One software (Bio-Rad). For pulse chase-analysis, 48 hours post-transfection cells were treated with 75 µg/ml cycloheximide (AG Scientific) freshly dissolved in water, or with the vehicle control for 0–6 hours, at which time cells were harvested in boiling SDS/DTT lysis buffer and electrophoresed for western blots. MG132 was purchased from Boston Biochem, dissolved in DMSO and used at [15 µM] final concentration. Statistical analyses were performed using the Student T-test. Immunofluorescence was conducted as previously described [29]. All images were taken with a 100X oil-immersion lens.

### Immunopurifications

Immunopurifications were conducted as previously described [29]–[31]. Briefly: for co-immunopurification, cells pelleted in ice-cold PBS were lysed in NETN lysis buffer ([50 mM] TRIS-pH 7.5, [150 mM] NaCl, 0.5% NP40) supplemented with protease inhibitor (PI) tablets (Sigma-Aldrich) and tagged protein was precipitated using anti-tag, bead-bound antibodies (Sigma-Aldrich). Beads were washed 3X with NETN+PI and protein was eluted from beads with 2% SDS.

For stringent, denature-renature immunopurification, cells were lysed in RIPA buffer ([50 mM] Tris, [150 mM] NaCl, 0.1% SDS, 0.5% deoxycholic-acid, 1% NP40, pH 7.4) + PI, denatured in 1% final concentration SDS for 30 minutes at room temperature and then renatured in 4.5% final concentration TritonX-100 for 30 minutes. Protein immunopurified with bead-bound antibodies (Sigma-Aldrich) was rinsed extensively with RIPA+PI (5X–8X) and eluted with 2% SDS.

## References

[1] Buchberger A, Bukau B, Sommer T (2010). Protein quality control in the cytosol and the endoplasmic reticulum: brothers in arms.. Mol Cell.

[2] Komander D, Clague MJ, Urbe S (2009). Breaking the chains: structure and function of the deubiquitinases.. Nat Rev Mol Cell Biol.

[3] Reyes-Turcu FE, Ventii KH, Wilkinson KD (2009). Regulation and cellular roles of ubiquitin-specific deubiquitinating enzymes.. Annu Rev Biochem.

[4] Nijman SM, Luna-Vargas MP, Velds A, Brummelkamp TR, Dirac AM (2005). A genomic and functional inventory of deubiquitinating enzymes.. Cell.

[5] Todi SV, Paulson HL (2011). Balancing act: deubiquitinating enzymes in the nervous system..

[6] Claessen JH, Kundrat L, Ploegh HL (2011). Protein quality control in the ER: balancing the ubiquitin checkbook..

[7] Kikkert M, Doolman R, Dai M, Avner R, Hassink G (2004). Human HRD1 is an E3 ubiquitin ligase involved in degradation of proteins from the endoplasmic reticulum.. J Biol Chem.

[8] Ballar P, Pabuccuoglu A, Kose FA (2011). Different p97/VCP complexes function in retrotranslocation step of mammalian ER-associated degradation (ERAD).. Int J Biochem Cell Biol.

[9] Bernardi KM, Williams JM, Kikkert M, van Voorden S, Wiertz EJ (2010). The E3 ubiquitin ligases Hrd1 and gp78 bind to and promote cholera toxin retro-translocation.. Mol Biol Cell.

[10] Raasi S, Wolf DH (2007). Ubiquitin receptors and ERAD: a network of pathways to the proteasome.. Semin Cell Dev Biol.

[11] Vembar SS, Brodsky JL (2008). One step at a time: endoplasmic reticulum-associated degradation.. Nat Rev Mol Cell Biol.

[12] Schmidt M, Hanna J, Elsasser S, Finley D (2005). Proteasome-associated proteins: regulation of a proteolytic machine.. Biol Chem.

[13] Elsasser S, Finley D (2005). Delivery of ubiquitinated substrates to protein-unfolding machines.. Nat Cell Biol.

[14] Ernst R, Mueller B, Ploegh HL, Schlieker C (2009). The otubain YOD1 is a deubiquitinating enzyme that associates with p97 to facilitate protein dislocation from the ER.. Mol Cell.

[15] Hassink GC, Zhao B, Sompallae R, Altun M, Gastaldello S (2009). The ER-resident ubiquitin-specific protease 19 participates in the UPR and rescues ERAD substrates.. EMBO Rep.

[16] Wang Q, Li L, Ye Y (2006). Regulation of retrotranslocation by p97-associated deubiquitinating enzyme ataxin-3.. J Cell Biol.

[17] Zhong X, Pittman RN (2006). Ataxin-3 binds VCP/p97 and regulates retrotranslocation of ERAD substrates.. Hum Mol Genet.

[18] Denuc A, Bosch-Comas A, Gonzalez-Duarte R, Marfany G (2009). The UBA-UIM domains of the USP25 regulate the enzyme ubiquitination state and modulate substrate recognition.. PLoS One.

[19] Meulmeester E, Kunze M, Hsiao HH, Urlaub H, Melchior F (2008). Mechanism and consequences for paralog-specific sumoylation of ubiquitin-specific protease 25.. Mol Cell.

[20] Bosch-Comas A, Lindsten K, Gonzalez-Duarte R, Masucci MG, Marfany G (2006). The ubiquitin-specific protease USP25 interacts with three sarcomeric proteins.. Cell Mol Life Sci.

[21] Kim I, Ahn J, Liu C, Tanabe K, Apodaca J (2006). The Png1-Rad23 complex regulates glycoprotein turnover.. J Cell Biol.

[22] Younger JM, Chen L, Ren HY, Rosser MF, Turnbull EL (2006). Sequential quality-control checkpoints triage misfolded cystic fibrosis transmembrane conductance regulator.. Cell.

[23] Nakatsukasa K, Huyer G, Michaelis S, Brodsky JL (2008). Dissecting the ER-associated degradation of a misfolded polytopic membrane protein.. Cell.

[24] Kim SM, Acharya P, Engel JC, Correia MA (2010). Liver cytochrome P450 3A ubiquitination in vivo by gp78/autocrine motility factor receptor and C terminus of Hsp70-interacting protein (CHIP) E3 ubiquitin ligases: physiological and pharmacological relevance.. J Biol Chem.

[25] Kaneko M, Koike H, Saito R, Kitamura Y, Okuma Y (2010). Loss of HRD1-mediated protein degradation causes amyloid precursor protein accumulation and amyloid-beta generation.. J Neurosci.

[26] Dantuma NP, Lindsten K, Glas R, Jellne M, Masucci MG (2000). Short-lived green fluorescent proteins for quantifying ubiquitin/proteasome-dependent proteolysis in living cells.. Nat Biotechnol.

[27] Li X, Zhao X, Fang Y, Jiang X, Duong T (1998). Generation of destabilized green fluorescent protein as a transcription reporter.. J Biol Chem.

[28] Berke SJ, Chai Y, Marrs GL, Wen H, Paulson HL (2005). Defining the role of ubiquitin-interacting motifs in the polyglutamine disease protein, ataxin-3.. J Biol Chem.

[29] Todi SV, Laco MN, Winborn BJ, Travis SM, Wen HM (2007). Cellular turnover of the polyglutamine disease protein ataxin-3 is regulated by its catalytic activity.. J Biol Chem.

[30] Todi SV, Scaglione KM, Blount JR, Basrur V, Conlon KP (2010). Activity and cellular functions of the deubiquitinating enzyme and polyglutamine disease protein ataxin-3 are regulated by ubiquitination at lysine 117.. J Biol Chem.

[31] Todi SV, Winborn BJ, Scaglione KM, Blount JR, Travis SM (2009). Ubiquitination directly enhances activity of the deubiquitinating enzyme ataxin-3.. EMBO J.

[32] Ernst R, Claessen JH, Mueller B, Sanyal S, Spooner E (2011). Enzymatic blockade of the ubiquitin-proteasome pathway.. PLoS Biol.

[33] Morreale G, Conforti L, Coadwell J, Wilbrey AL, Coleman MP (2009). Evolutionary divergence of valosin-containing protein/cell division cycle protein 48 binding interactions among endoplasmic reticulum-associated degradation proteins.. FEBS J.

[34] Denuc A, Marfany G (2010). SUMO and ubiquitin paths converge.. Biochem Soc Trans.

[35] Scaglione KM, Zavodszky E, Todi SV, Patury S, Xu P (2011). Ube2w and Ataxin-3 Coordinately Regulate the Ubiquitin Ligase CHIP.. Mol Cell.

[36] Sowa ME, Bennett EJ, Gygi SP, Harper JW (2009). Defining the human deubiquitinating enzyme interaction landscape.. Cell.

[37] Ventii KH, Wilkinson KD (2008). Protein partners of deubiquitinating enzymes.. Biochem J.

[38] Goate A, Hardy J (2012). Twenty years of Alzheimer's disease-causing mutations.. J Neurochem.

[39] Tresse E, Salomons FA, Vesa J, Bott LC, Kimonis V (2010). VCP/p97 is essential for maturation of ubiquitin-containing autophagosomes and this function is impaired by mutations that cause IBMPFD.. Autophagy.

[40] Williams AJ, Knutson TM, Colomer Gould VF, Paulson HL (2009). In vivo suppression of polyglutamine neurotoxicity by C-terminus of Hsp70-interacting protein (CHIP) supports an aggregation model of pathogenesis.. Neurobiol Dis.

[41] Valero R, Bayes M, Francisca Sanchez-Font M, Gonzalez-Angulo O, Gonzalez-Duarte R (2001). Characterization of alternatively spliced products and tissue-specific isoforms of USP28 and USP25.. Genome Biol.

[42] Valero R, Marfany G, Gonzalez-Angulo O, Gonzalez-Gonzalez G, Puelles L (1999). USP25, a novel gene encoding a deubiquitinating enzyme, is located in the gene-poor region 21q11.2.. Genomics.

